# Fenestration in the P1 Segment of the Posterior Cerebral Artery

**DOI:** 10.7759/cureus.1528

**Published:** 2017-07-31

**Authors:** Chad J Jensen, Rafik Shereen, R. Shane Tubbs, Christoph Griessenauer

**Affiliations:** 1 Department of Surgery, The Brooklyn Hospital Center; 2 Department of Anatomical Sciences, St. George's University School of Medicine, Grenada, West Indies; 3 Neurosurgery, Seattle Science Foundation; 4 Neurosurgery Service, Department of Surgery, Beth Israel Deaconess Medical Center, Harvard Medical School

**Keywords:** fenestration, posterior cerebral artery, p1 segment

## Abstract

The posterior cerebral artery (PCA) has been noted in literature to have anatomical variations, specifically fenestration. Cerebral arteries with fenestrations are uncommon, especially when associated with other vascular pathologies. We report a case here of fenestrations within the P1 segment of the right PCA associated with a right middle cerebral artery (MCA) aneurysm in an elder adult male who presented with a new onset of headaches. The patient was treated with vascular clipping of the MCA and has recovered well. Identifying anatomical variations with appropriate imaging is of particular importance in neuro-interventional procedures as it may have an impact on the procedure itself and consequently post-interventional outcomes.

## Introduction

The basilar artery bifurcates at its superior terminus, yielding the right and left posterior cerebral arteries. Anatomically, the posterior cerebral artery (PCA) is divided into four segments, P1-P4. While there is some disagreement on the classification of certain segments, all classification systems hold that P1 is from the origin of the PCA to the anastomosis with the posterior communicating artery (PCoA). The P1, also known as the pre-communicating segment, resides within the interpeduncular cistern and gives off the following branches: the posterior thalamoperforating artery, direct perforating artery, long circumflex artery, and short circumflex artery [1-2].

Multiple variations of the PCA such as fenestration, early bifurcation, and duplication have been reported in past literature. Bayrak, et al. define a fenestration as “a vascular variation that begins with a common origin, then splits into two parallel luminal channels and rejoins distally" [3]. There have only been a handful of cases in the literature that describe fenestrations within the P1 segment, and from 1969 to 2014 there were only 19 reported cases [1]. Since then, researchers have sought to use computed tomography (CT) angiography to identify variations since in the past most have been discovered only through cadaveric dissection [4]. Herein, we report a rare case of P1 fenestration identified on magnetic resonance angiography (MRA).

## Case presentation

**Figure 1:**
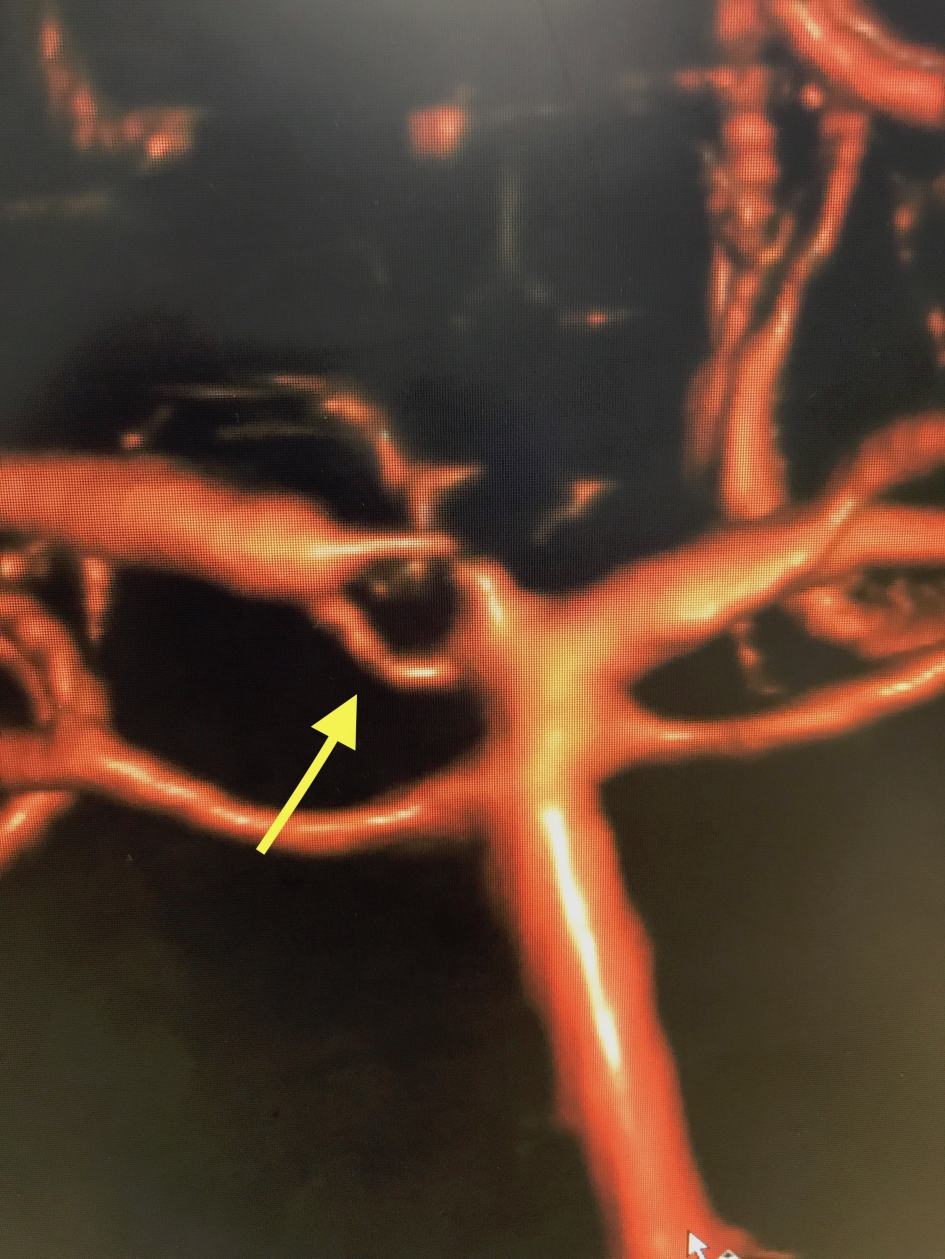
Here we see a bifurcation of the basilar artery into left and right posterior cerebral arteries. Note the fenestration of the P1 segment of the right posterior cerebral artery highlighted by the yellow arrow.

A 56-year-old Caucasian male presented with new onset of headaches and underwent a screening MRA. Imaging identified a right middle cerebral artery (MCA) aneurysm. The patient underwent a subsequent diagnostic digital subtraction angiogram that confirmed the MCA aneurysm measuring 4 mm in maximum diameter. He was also found to have fenestration of the right P1 segment of the PCA (Figure 1). This fenestration was not apparent on the preceding MRA. There was a vessel drop out on MRA in that area of the PCA, but no fenestration was visualized. The patient underwent uneventful clipping of the MCA aneurysm and is doing well at last follow-up. No additional intracranial anomalies were identified.

## Discussion

Fenestration of the P1 segment of the PCA is of particular importance for neuro-intervention. The literature is well documented with cases of aneurysms within the PCA and specifically the P1 segment with concomitant fenestration. It is, therefore, important to be aware of anatomical variations in the artery, as presented in this case report.

In a review of the literature, there are multiple variations of the PCA such as fenestration, early bifurcation, and duplication. Vlajković, et al. examined the PCA of 468 (200 fetal and 268 adults) cadavers, finding four cases (0.85%) of fenestration with two of them being in the P1 segment [1]. In their study, they surveyed the literature for similar cases of fenestration within the PCA. The earliest case dated to 1969 and the authors found a total of 19 cases, which included their own contributions. There was a total of 11 fenestrations occurring within the P1 segment. Caruso, et al. studied the P1 segment in 100 human fixed brains and found that its length ranged from 2.5 to 14 mm with an average of 6.7 +/- 2.6 mm, with a caliber ranging from 1 to 4, an average of 2.3 +/- 0.7 mm [5]. Their study also reported three fenestrations all within the P1 segment, suggesting that fenestrations have a predilection for the P1 segment [5].

Uchino, et al. published a study on the prevalence of the aforementioned PCA variations [4]. The authors had reviewed MR angiographic images of 2350 patients and found that fenestration was present in only eight (0.34%) cases. The fenestrations were also mostly found within the P1 segment and also at the P1-P2 junction. In evaluating the prevalence of fenestrations in intracranial arteries using CT angiography Bayrak, et al. found in their study of 438 patients that the most common site is within the vertebrobasilar system [3]. Within that system, there were 22 cases yielding an overall rate of 5.56%, compared to two cases (0.05%) of fenestration found in the PCA. Fenestrations within the PCA, therefore, are truly a rare finding.

## Conclusions

The present case report provides a variation of the PCA regarding fenestrations within the region of the P1 branch. With the importance of the PCA morphology, symmetry, and branching patterns this case provided an opportunity to dive into previous research to help classify the prevalence of such a variant and provided an example of a well-identified fenestration in the P1 segment of the PCA with MRA. While past literature suggests that fenestrations of the PCA remain an uncommon finding, we hope these findings can aid in future identification and awareness of the different variants of the PCA for neurological intervention.

## References

[1] Vlajković S, Vasović L, Trandafilović M (2015). Fenestrations of the human posterior cerebral artery. Childs Nerv Syst.

[2] Seoane ER, Tedeschi H, de Oliveira E (1997). Management strategies for posterior cerebral artery aneurysms: a proposed new surgical classification. Acta Neurochir.

[3] Bayrak AH, Senturk S, Akay HO (2011). The frequency of intracranial arterial fenestrations: a study with 64-detector CT-angiography. Eur J Radiol.

[4] Uchino A, Saito N, Takahashi M (2016). Variations of the posterior cerebral artery diagnosed by MR angiography at 3 tesla. Neuroradiology.

[5] Caruso G, Vincentelli F, Rabehanta P (1991). Anomalies of the P1 segment of the posterior cerebral artery: early bifurcation or duplication, fenestration, common trunk with the superior cerebellar artery. Acta Neurochir.

